# From Europe

**Published:** 1872-09

**Authors:** C. M. Wright


### CORRESPONDENCE.

### From Europe.

Mr. Editor.—The last thing you said to me was: "You must send us something for the Register from Europe." I can not disobey such a command. Now shall I try to be wise and write descriptions of imaginary European experiences, or shall I write right on, like an honest Ohio dentist, who does not care a copper whether he is considered wise or not, if you'll only believe him honest? The latter plan will be the most natural. Well for the trip. To cross the old Atlantic in a stauch Cunarder is grand. The sensations are a healthy exhileration; a robust feeling of *hurrah*—provided you are not sea-sick and have an appetite for your four meals a day, and your regular allowance of tobacco, as I had, for about six days. Then you get tired; the excitement is over; the sea becomes monotonous, and you want a little terra firma—even a flower pot-full of mother earth is pleasant to look upon. If a squall comes, however, your spirits rise, and to sit or stand at the stern of the vessel and see the great waves come on and the ship bury her bow in water, and then rise as though she had determined to fly toward heaven; up and down, from side to side. By George it's grand. Every body sick below stairs; but your non-bilious condition, or your non-sea-sick condition keeping you well and jolly; you enjoy this hugely. Nevertheless when the picturesque coast of "ould Ireland" appears you are as happy as a clam though not so cool. At Queenstown you say good-bye to your friends; Parsons who is literary, and has written some interesting papers for the *Atlantic Monthly*; a Pittsburgh gentleman who is an old traveler, and others whom you have learned to regard as part of your company on board. Next day comes Liverpool with the finest docks in the world; then London—grand, old, glorious London. You feel as though you were at home, so many of the places seem so familar; and you instictively look for your old friend Addison, Swift and Johnson, Thackery, Bulwer, and Charles Dickens. The English Channel is dreaded more than the Atlantic, for every body has told you, "Well, you may get over the Atlantic without sea-sickness, but you are bound to succumb to the chopping waves of the Channel." Well, now you are on the small tug steamer on the Channel. Stewards are running about with wash-bowls, and passengers are groaning and heaving. You select your steward and bide your time. An hour passes; you are in the middle of the channel, and can see "la belle France." The sea is rough, but sea sickness don't come. Aha! "Steward bring me a bottle of ale and some sandwiches." You drink your ale on deck, and poor miserable passengers look on you with eyes filled with disgust, but only for a minute, the sight of the ale causes them to hold sudden and confidential conversations with the bowls before them, and the stewards hasten to hold their heads and perform other kind offices. Bah! why are they sea-sick? Next comes Paris—beautiful Paris—with her marks of the incendiary Commune—grand old piles of ruins, splendid buildings destroyed by fire, and on the buildings are signs, "Equalite, Fraternite, Liberte." The beautiful Champs Elysees is on ordinary days a paradise for idle, pleasure seekers, children and loafers. Nothing an Ohio man has ever seen equals this one grand avenue—concert halls, flying Dutchmen, French Punch and Judy shows, goat teems, toy booths, gaily dressed woman, fancily dressed and playful children, happy Frenchmen, joyful promenaders—well hurrah for the Champs Elysees. After all this glory, this charm of travel, this surfeit of sight seeing, this excitement of new scenes and pleasures, when every day is a week, and when every night finds your eyes sticking out of your head like a snail's—after all this comes beautiful Switzerland. And then daily dental practice—filling teeth in this country; and when just out of sight of your operating window can be seen the everlasting snow mountains; is just as hard work, and makes the back ache just the same as in Cincinnati, with her sable mantel of coal soot. In a very few weeks the charm of travel is like a dream that is past, and work, work, becomes the daily business; and Oxford, Riley, Camden, or Cincinnati are just as beautiful places, just as comfortable, from which to view oral cavities. There is the same odor of iodine, and carbolic acid in an office in Switzerland as in Cincinnati.

The same odor escapes from pulp cavities from the decomposed defunct pulps. People say "Mein Gott" instead of "Geeminy Whilikers" when you cut sensitive dentine. In fact business is business here or there or anywhere. You can't escape the petty annoyances—there is no Champs Elysees for dentists. Then in Switzerland the quality of the teeth seems not so good even as in America. Irregularities abound, and singularities so regarded in America are common here. Soft, chalky teeth, dentine and enamel are common, and are affected with the destructive white and light brown colored caries. Then the disease that affects the necks of teeth, wasting away enamel and dentine, as though you had taken Morrison's engine and a barrel shaped burr and cut into the necks of the teeth; leaving the surface smooth, white, and apparently as hard as the rest of the teeth, is common. Salivary calculus is quite a common disease too. I haven't been here long enough yet to have a theory about the cause or causes of the diseases of the teeth. Theories are good things you know, Mr. Editor. You remember the Irish doctor who had a theory about a disease of the stomach. He said: "I belave docther the mon had wather on the chest, and the wather keeps a rotten' and a rotten' up again the diaphram till it rotted a hole in it, and then rushed in and drowned out the lungs, and the mon died." I am only telling you how I find things. I am using the Morrison engine, and like it very much. For finishing fillings, for cutting out old amalgam fillings, (we have that to do here; for native dentists will put in a poor quality of amalgam even in fine-looking incisors) for preparing cavities, after the enamel chisel, for polishing teeth, for opening up pulp cavities after the Hullihen method (we have that to do here too), and for a labor saving, back saving, good instrument, it is invaluable. But I will stop. I've got lots more to say, but must not burden your journal too much. I've tried to avoid what Dr. Driscoll calls the sensational newspaper style; but my pen has had several jolly frolics on a separate sheet. But as I am no longer professor, perhaps the influence for evil (God help us) is not so great, and even Dr. Driscoll may forgive a fellow for writing as naturally as he thinks and talks without a bit of the sham dignity of shallow headed fellows, who wear a number 6 hat, and must take care of their moiety of brains. Tell me whether I shall write again. C. M. WRIGHT.

